# Effects of Substitution on Cytotoxicity of Diphenyl Ditelluride in Cultured Vascular Endothelial Cells

**DOI:** 10.3390/ijms221910520

**Published:** 2021-09-29

**Authors:** Takato Hara, Takahiro Okazaki, Tamayo Hashiya, Kyohei Nozawa, Shuji Yasuike, Jyoji Kurita, Chika Yamamoto, Noriaki Hamada, Toshiyuki Kaji

**Affiliations:** 1 Faculty of Pharmaceutical Sciences, Toho University, 2-2-1 Miyama, Funabashi 274-8510, Japan; takato.hara@phar.toho-u.ac.jp (T.H.); yamamoto@phar.toho-u.ac.jp (C.Y.); 2 Faculty of Pharmaceutical Sciences, Tokyo University of Science, 2641 Yamazaki, Noda 278-8510, Japan; 3b10032@alumni.tus.ac.jp (T.O.); 3a08096@alumni.tus.ac.jp (T.H.); 3 Faculty of Science and Technology, Tokyo University of Science, 2641 Yamazaki, Noda 278-8510, Japan; kyoh.nozawa@gmail.com; 4 School of Pharmaceutical Sciences, Aichi Gakuin University, 1-100 Kusumoto-cho, Chikusa-ku, Nagoya 464-8650, Japan; s-yasuik@dpc.agu.ac.jp; 5 Faculty of Pharmaceutical Sciences, Hokuriku University, Ho-3 Kanagawa-machi, Kanazawa 920-1181, Japan; jyomarronta205@gmail.com

**Keywords:** bio-organometallics, cytotoxicity, organotellurium compound, organoselenium compound

## Abstract

Among organic–inorganic hybrid molecules consisting of organic structure(s) and metal(s), only few studies are available on the cytotoxicity of nucleophilic molecules. In the present study, we investigated the cytotoxicity of a nucleophilic organotellurium compound, diphenyl ditelluride (DPDTe), using a cell culture system. DPDTe exhibited strong cytotoxicity against vascular endothelial cells and fibroblasts along with high intracellular accumulation but showed no cytotoxicity and had less accumulation in vascular smooth muscle cells and renal epithelial cells. The cytotoxicity of DPDTe decreased when intramolecular tellurium atoms were replaced with selenium or sulfur atoms. Electronic state analysis revealed that the electron density between tellurium atoms in DPDTe was much lower than those between selenium atoms of diphenyl diselenide and sulfur atoms of diphenyl disulfide. Moreover, diphenyl telluride did not accumulate and exhibit cytotoxicity. The cytotoxicity of DPDTe was also affected by substitution. *p*-Dimethoxy-DPDTe showed higher cytotoxicity, but *p*-dichloro-DPDTe and *p*-methyl-DPDTe showed lower cytotoxicity than that of DPDTe. The subcellular distribution of the compounds revealed that the compounds with stronger cytotoxicity showed higher accumulation rates in the mitochondria. Our findings suggest that the electronic state of tellurium atoms in DPDTe play an important role in accumulation and distribution of DPDTe in cultured cells. The present study supports the hypothesis that nucleophilic organometallic compounds, as well as electrophilic organometallic compounds, exhibit cytotoxicity by particular mechanisms.

## 1. Introduction

Organic–inorganic hybrid molecules such as metal complexes and organometallic compounds were first developed as chemical synthesis reagents [1] and are now used not only in synthetic chemistry and catalysis science but also in materials science. We have proposed and reported the effects of organic–inorganic hybrid molecules on biological systems as bio-organometallics [2]. In particular, controlling the cytotoxicity of organic–inorganic hybrid molecules is one of the most important requirements for ensuring safe use of such compounds. Our previous study indicated that the electronic state of the compounds is crucial for cytotoxicity of organic–inorganic hybrid molecules [3,4,5]. In fact, many inorganic and organometallic compounds that have been studied for cytotoxicity are mainly electrophilic and have shown a high affinity for nucleophilic molecules in cells. Thus, biological effects of electrophilic organometallic compounds have been elucidated [6,7,8,9,10], but little is known regarding the biological effects of nucleophilic organometallic compounds. We focused on diphenyl ditelluride (DPDTe), a nucleophilic organotellurium compound because toxicological research on chalcogen compounds is limited and DPDTe is a small molecule that is easy to handle. Although there is little opportunity for human exposure to this compound, DPDTe is an excellent tool to study the toxicological mechanisms of nucleophilic organometallic compounds. This study aimed to establish the relationship between cytotoxicity and electronic state of nucleophilic hybrid molecules using DPDTe and its substituted compounds.

## 2. Results

### 2.1. Diphenyl Ditelluride (DPDTe) Accumulates and Exhibits Cytotoxicity in Vascular Endothelial Cells and Fibroblastic IMR-90 Cells

**Figure 1:**
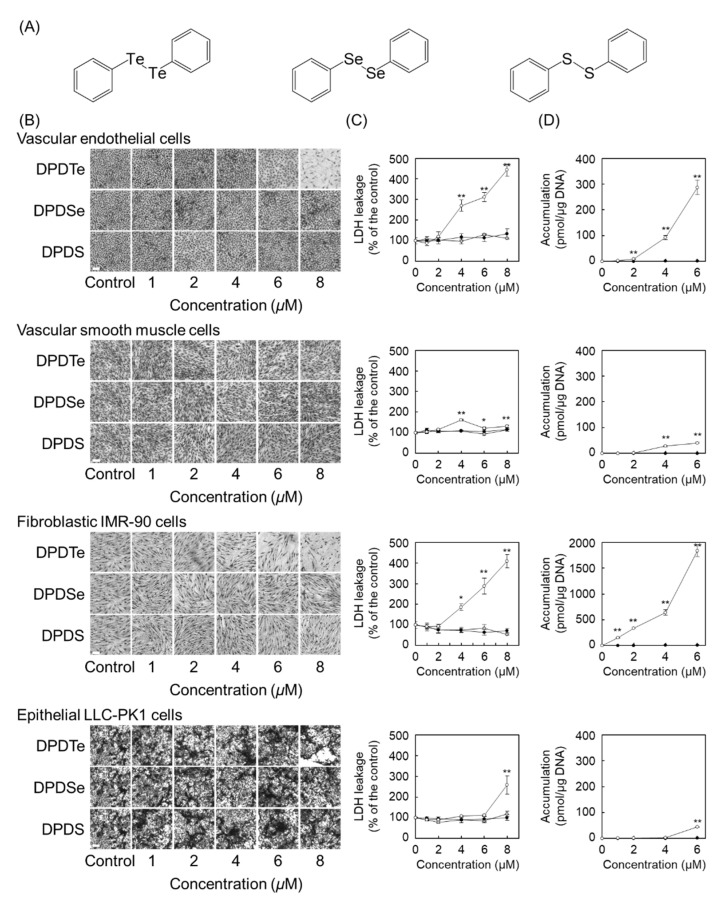
Cytotoxicity and intracellular accumulation of diphenyl ditelluride (DPDTe), and compounds, in which tellurium in DPDTe was replaced by selenium and sulfur atoms (DPDSe and DPDS). Vascular endothelial cells, vascular smooth muscle cells, fibroblastic IMR-90 cells, and epithelial LLC-PK1 cells were treated with DPDTe, DPDSe, or DPDS (1, 2, 4, 6, or 8 µM) for 24 h. (**A**) Structure of DPDTe (left), DPDSe (middle), and DPDS (right). (**B**) Morphological observation (bar = 50 µm) and (**C**) lactate dehydrogenase (LDH) leakage from the cells treated with DPDTe (white), DPDSe (black), or DPDS (gray). (**D**) Intracellular accumulation of DPDTe (white) and DPDSe (black). Values are expressed as the mean ± standard error of three replicates. * *p* < 0.05, ** *p* < 0.01 compared with the corresponding control.

We first investigated whether DPDTe (Figure 1A) exhibits cytotoxicity in vascular endothelial cells, vascular smooth muscle cells, fibroblastic IMR-90 cells, and epithelial LLC-PK1 cells. Morphological observation revealed that DPDTe exhibited cytotoxicity with disruption of the cell layer in a dose-dependent manner against vascular endothelial cells and fibroblastic IMR-90 cells, but not vascular smooth muscle cells and epithelial LLC-PK1 cells. Conversely, the compounds, in which tellurium in DPDTe was replaced by selenium and sulfur atoms (DPDSe and DPDS, respectively), did not show marked morphological changes in the cells used in this study (Figure 1B). The cytotoxicity of these compounds was quantified by measuring the leakage of lactate dehydrogenase (LDH) from the cells. Similar to the morphological appearance, LDH leakage was significantly increased only in vascular endothelial cells and fibroblastic IMR-90 cells exposed to DPDTe (Figure 1C). Since intracellular accumulation is an indicator of the extent of cytotoxicity of organic–inorganic hybrid molecules, we analyzed the accumulation pattern of DPDTe and DPDSe in the cells. DPDTe was highly accumulated in vascular endothelial cells and fibroblastic IMR-90 cells, but less accumulation was observed in vascular smooth muscle cells and epithelial LLC-PK1 cells. However, DPDSe did not accumulate in any of the cells tested (Figure 1D).

### 2.2. Cleavage of Te–Te Bond Is Important for Accumulation and Cytotoxicity of DPDTe

**Figure 2:**
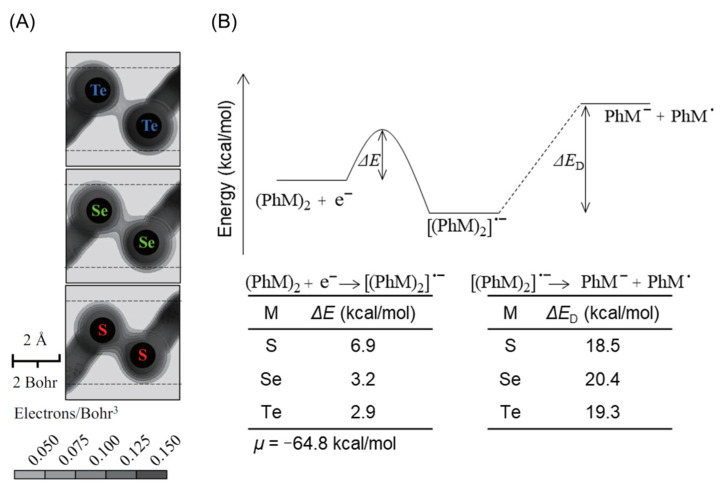
Estimated cleavage process of DPDTe, DPDSe, and DPDS. (**A**) Electron density of DPDTe, DPDSe, and DPDS determined by ab initio quantum chemical calculations. The darker the black, the higher the electron density. (**B**) Energy diagram of DPDTe, DPDSe, and DPDS in heterolytic cleavage process.

To further elucidate the underlying reason of cytotoxicity associated with DPDTe, we analyzed the electronic states of the molecules. Ab initio quantum chemical calculations were performed using the GAMESS [11]. The electron density between tellurium atoms in DPDTe was much lower than those of selenium atoms in DPDSe and sulfur atoms in DPDS. Additionally, the interatomic distance of tellurium atoms in DPDTe was longer than those of selenium atoms in DPDSe and sulfur atoms in DPDS (Figure 2A). Homolytic cleavage of the neutral DPDM (1) costs a large amount of energy: 54.6, 50.1, and 47.2 kcal/mol for M = S, Se, and Te, respectively.

(PhM)_2_ → 2PhM^•^   (M = S, Se, Te) (1)

We examined another reaction path with a heterolytic cleavage (Figure 2B).

(PhM)_2_ + e^−^ → [(PhM)_2_]^•^^−^ → PhM^•^ + PhM^−^   (M = S, Se, Te), (2)

where (PhM)_2_ gets an electron, and then dissociates into PhM^•^ radical and PhM^−^ ion. To complete the energy diagram of this process, we need to know the chemical potential of electrons on the initial electron transfer to (PhM)_2_. Antonello et al. have extensively investigated the dissociation process of various disulfides and found that the initial electron transfer needs activation energy, which was estimated to be 6.9 kcal/mol for (PhS)_2_ from an electrochemical experiment [12]. In our calculation, the value of activation energy was obtained by setting *μ* = −64.8 kcal/mol as the chemical potential of the electron. After adopting the same value as *μ* for (PhM)_2_, we obtained activation energies of 3.2 and 2.9 kcal/mol for M = Se and Te, respectively. DPDTe was identified as the most easily ionized molecule among all. The second cleavage reaction costs a small amount of energy: 18.5, 20.4, and 19.3 kcal/mol for M = S, Se, and Te, respectively. Thus, we found that heterolytic cleavage easily occurs in DPDTe compared to DPDS and DPDSe.

These values show that the heterolytic cleavage through radical anionic molecule (2) occurs much more easily than the homolytic cleavage in neutral molecule (1). Furthermore, process (2) may be followed by:

PhM^•^ + e^−^ → PhM^−^   (M = S, Se, Te) (3)

After combining the reactions:

(PhM)_2_ + 2e^−^ → 2PhM^−^   (M = S, Se, Te) (4)

The heterolytic cleavage through [(PhTe)_2_]^•^^−^ is a key process that promotes the cleavage itself and also produces the anions PhTe^−^.

**Figure 3:**
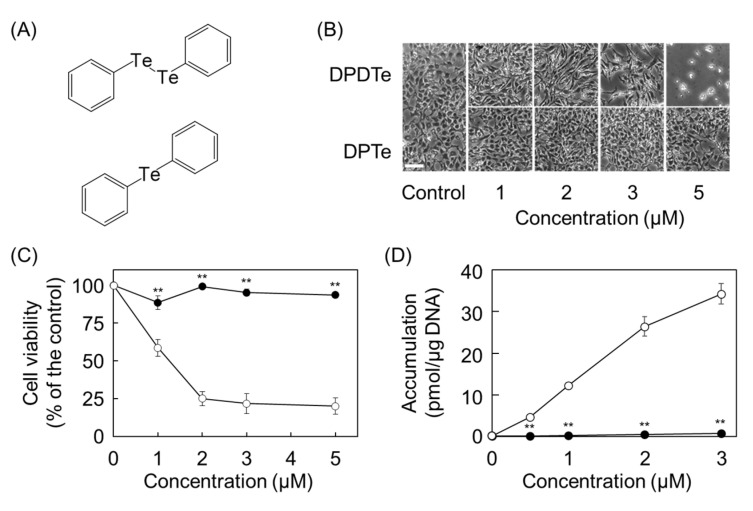
Assessment of contribution of the Te–Te bond to cytotoxicity of DPDTe. (**A**) Structure of DPDTe (top) and DPTe (bottom). (**B**) Morphological observation (bar = 50 µm), (**C**) viability of vascular endothelial cells treated with DPDTe (white) and DPTe (black), and (**D**) intracellular accumulation of DPDTe (white) and DPTe (black) in vascular endothelial cells after treatment (0.5, 1, 2, 3, or 5 µM) for 24 h. Values are expressed as the mean ± standard error of three replicates. ** *p* < 0.01 compared with the corresponding DPDTe treatment.

Since these results suggest that the cleavage of the Te–Te bond is a key factor that determines the cytotoxicity of DPDTe, we synthesized stable compound diphenyl telluride (DPTe) (Figure 3A) and analyzed its cytotoxicity in vascular endothelial cells. DPTe did not affect the morphology and viability of vascular endothelial cells (Figure 3B,C), and it was scarcely accumulated in the cells (Figure 3D).

### 2.3. Substitution Modulate Cytotoxicity of DPDTe

**Figure 4:**
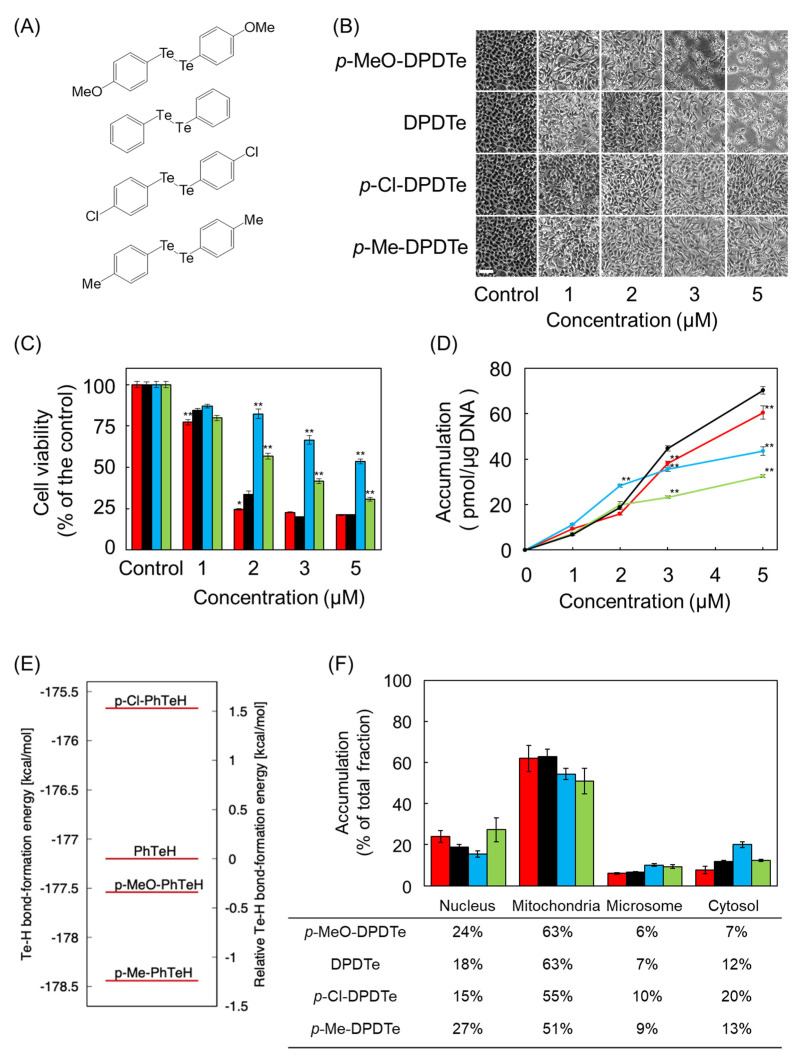
Effect of substitution on cytotoxicity and intracellular accumulation of DPDTe. (**A**) Structure of *p*-MeO-DPDTe, DPDTe, *p*-Cl-DPDTe, and *p*-Me-DPDTe. (**B**) Morphological observation (bar = 50 µm), (**C**) viability of vascular endothelial cells, and (**D**) intracellular accumulation of *p*-MeO-DPDTe, DPDTe, *p*-Cl-DPDTe, and *p*-Me-DPDTe. Vascular endothelial cells were treated with *p*-MeO-DPDTe (red), DPDTe (black), *p*-Cl-DPDTe (blue), and *p*-Me-DPDTe (green) (1, 2, 3, or 5 µM) for 24 h. (**E**) Te–H bond formation energies in *p*-MeO-PhTeH, PhTeH, *p*-Cl-PhTeH, and *p*-Me-PhTeH. (**F**) Accumulation of *p*-MeO-DPDTe, DPDTe, *p*-Cl-DPDTe, and *p*-Me-DPDTe in cellular fractions. Vascular endothelial cells were treated with 2 µM of *p*-MeO-DPDTe (red), DPDTe (black), *p*-Cl-DPDTe (blue), and *p*-Me-DPDTe (green) for 24 h. Values are expressed as the mean ± standard error of three replicates. * *p* < 0.05, ** *p* < 0.01 compared with the corresponding DPDTe treatment.

Next, we examined the effect of substitution on cytotoxicity by using *p*-dimethoxy-DPDTe (*p*-MeO-DPDTe), *p*-dichloro-DPDTe (*p*-Cl-DPDTe), and *p*-dimethyl-DPDTe (*p*-Me-DPDTe), in which electron-donating or electron-withdrawing groups were added to DPDTe (Figure 4A). Morphological observation and cell viability assays revealed that *p*-MeO-DPDTe showed higher cytotoxicity than that of DPDTe. Conversely, *p*-Cl-DPDTe and *p*-Me-DPDTe showed lower cytotoxicity than that of DPDTe (Figure 4B,C). However, the extent of cytotoxicity could not be explained only by the intracellular accumulation of these compounds (Figure 4D). To determine the underlying reason for cytotoxicity of these compounds, we calculated the Te–H bond formation energy in the protonation of these compounds. The calculated bond formation energy could be related to cytotoxicity in *p*-MeO-PhTeH, PhTeH, and *p*-Cl-PhTeH, except *p*-Me-PhTeH. This was identified as a useful indicator of cytotoxicity (Figure 4E and Table S1), suggesting that substitution affected the mechanism of cytotoxicity of these compounds after their uptake into the cells. To test this hypothesis, vascular endothelial cells were fractionated, and the amount of tellurium accumulated in each fraction was measured. As shown in Figure 4F, compounds with strong cytotoxicity showed higher accumulation in mitochondria and lower accumulation in microsomes and cytosol.

## 3. Discussion

In this study, we investigated the importance of the electronic state of the Te–Te bond on cytotoxicity of DPDTe, a simple nucleophilic organotellurium compound, by using its substituents. In general, the cytotoxicity of a compound is classified based on its accumulation in the cells. We have reported that this classification can be applied to cytotoxicity of organic–inorganic hybrid molecules [3,4,5,13,14]. Since DPDTe was highly accumulated and showed cytotoxicity in vascular endothelial cells and fibroblastic IMR-90 cells, DPDTe was classified as a type of molecule in which accumulation and cytotoxicity were correlated. However, DPDTe did not show cytotoxicity and scarcely accumulated in vascular smooth muscle cells and epithelial LLC-PK1 cells. Since we have reported that organic–inorganic hybrid molecules show cell type-dependent cytotoxicity [15], it was suggested that DPDTe has a cell-type dependent uptake mechanism.

Heterolytic cleavage through [(PhTe)_2_]^•^^−^ is a key process that promotes the cleavage itself and also produces PhTe^−^. Above all, dissociation of the Te–Te bond is important for the accumulation of DPDTe in vascular endothelial cells. This was supported by the finding that stable organotellurium compound DPTe did not accumulate in vascular endothelial cells. Similarly, DPDSe and DPDS did not accumulate and show cytotoxicity in any of the cells tested in this study. In addition, DPDTe accumulated in vascular endothelial cells and fibroblastic IMR-90 cells despite the lowest CLogP values among DPDTe, DPDSe, and DPDS (see Materials Section). This result also supports that DPDTe is dissociated and then taken up into these cells. Although there are no reports on DPDTe, several studies have shown that DPDSe is reduced by thioredoxin reductase [16,17]. In addition, the results showed that DPDTe can be more easily reduced than DPDSe. Therefore, it is conceivable that PhTe^−^, which is produced by the reduction of DPDTe outside the cells or on the cell surface, is taken up into the cells using molecules on the cell membrane, transporters, and channels, and that PhTe^−^ is the actual state of nucleophilic reactivity of DPDTe.

Moreover, the effect of substitution on DPDTe was investigated using *p*-MeO-DPDTe, *p*-Cl-DPDTe, and *p*-Me-DPDTe. The cytotoxicity was strongest for *p*-MeO-DPDTe, followed by DPDTe, *p*-Me-DPDTe, and then *p*-Cl-DPDTe. Except for *p*-Me-DPDTe, this order is consistent with the Te–H bond formation energy of the tellurate ion produced after cleavage of these organotellurium compounds. A possible reason for the lack of correlation between the cytotoxicity and intensity of the Te–H bond formation energy in *p*-Me-DPDTe is that the compound was less accumulated in vascular endothelial cells, particularly in their mitochondrial fraction, than other compounds. The detailed mechanism is not known. However, we reported that the cytotoxicity of some organometallic compounds is reduced by introducing a methyl group [5]; it is possible that this effect is applied to *p*-Me-DPDTe. Thus, substitution on the phenyl groups may play an important role in the cytotoxicity of these compounds by affecting the accumulation in the mitochondrial fraction and the reactivity of their anions.

It has been reported that DPDTe inhibits NADH quinone oxidoreductase, a component of the mitochondrial electron transport chain, by reacting with mitochondrial fraction from rat liver and kidney homogenates [18] and induces cell cycle arrest and apoptosis by inhibiting topoisomerase I in Chinese hamster fibroblast V79 cells [19]. These findings suggest mitochondria as a target organelle for DPDTe cytotoxicity. This study provides valuable findings by examining the cytotoxicity of nucleophilic organometallic compounds at the electron level and clarifies that the electronic state of these compounds contributes to the cellular response.

## 4. Materials and Methods

### 4.1. Materials

Bovine aortic endothelial cells and bovine aortic smooth muscle cells were purchased from Cell Applications (San Diego, CA, USA). Human fetal lung fibroblastic IMR-90 and porcine kidney epithelial LLC-PK1 cells were obtained from DS Pharma Biomedical (Osaka, Japan). AlamarBlue^®^ reagent was purchased from Thermo Scientific (Waltham, MA, USA). Dulbecco’s modified Eagle’s medium (DMEM) and Ca^2+^- and Mg^2+^-free phosphate-buffered saline (CMF-PBS) were obtained from Nissui Pharmaceutical (Tokyo, Japan). Tissue culture dishes and plates were obtained from AGC Techno Glass (Shizuoka, Japan). CytoTox 96 Non-Radioactive Cytotoxicity Assay kit was purchased from Promega (Madison, WI, USA). Other reagents of the highest grade available were obtained from Nacalai Tesque (Kyoto, Japan). DPDS was purchased from Tokyo Chemical Industry (Tokyo, Japan). DPDTe, DPDSe, *p*-MeO-DPDTe, *p*-Cl-DPDTe, *p*-Me-DPDTe and DPTe were synthesized as previously reported [20,21]. The CLogP of DPDTe, DPDSe, and DPDS calculated by ChemDraw Professional 19.0 (PerkinElmer, Waltham, MA, USA) were 3.19, 4.41, and 4.44, respectively.

### 4.2. Cell Culture and Treatments

Vascular endothelial and smooth muscle cells, fibroblastic IMR-90, and epithelial LLC-PK1 cells were cultured in a humidified atmosphere of 5% CO_2_ at 37 °C in DMEM supplemented with 10% fetal bovine serum until confluent. The cells were then transferred into either 6- or 24-well culture plates or 100 mm dishes at 2 × 10^4^ cells/cm^2^ and the experiments were performed as described below.

### 4.3. Cytotoxicity Assay and Cell Viability Assay

A cytotoxicity assay was performed as previously described [3]. Briefly, confluent vascular endothelial cells, vascular smooth muscle cells, fibroblastic IMR-90 cells, and epithelial LLC-PK1 cells grown in 24-well culture plates were treated with DPDTe, DPDSe, and DPDS for 24 h. After incubation, a portion of the treated medium was collected, and LDH activity was measured, as a marker for cell death, using the CytoTox 96 Non-Radioactive Cytotoxicity Assay kit. To analyze cell viability, mitochondrial activity was measured using a previously reported method with modification [22]. Confluent vascular endothelial cells grown in 24-well culture plates were treated with organotellurium compounds for 24 h. The conditioned media were discarded, and DMEM-AlamarBlue (10:1) solution was added to each well, and the cells were further incubated for 2 h. After incubation, DMEM-AlamarBlue was transferred from each well to a black bottom 96-well plate and fluorescence was measured (Ex = 544 nm, Em = 590 nm) using a multimodal plate reader (BMG LABTECH, Ortenberg, Germany).

### 4.4. Intracellular Accumulation of Organotellurium and Organoselenium Compounds

Confluent vascular endothelial cells, vascular smooth muscle cells, fibroblastic IMR-90 cells, and epithelial LLC-PK1 cells grown in 6-well plates were treated with organotellurium or organoselenium compounds for 24 h. After incubation, the cells were lysed and sampled for analysis of the tellurium and selenium atoms according to our previous report [5]. The conditions of inductively coupled plasma mass spectrometry (ICP-MS) (Nexion 300S, PerkinElmer) were optimized for plasma output of 1600 W, plasma gas flow rate of 18.0 L/min, and nebulizer gas flow rate of 0.94 L/min. Another portion of the cell lysate was analyzed for DNA content by the fluorometric method [23] to express the metal content as pmol/µg DNA.

For the cellular fraction, confluent vascular endothelial cells grown in 100 mm dishes were treated with organotellurium compounds for 24 h. After treatment, the conditioned media were discarded, and the cells were washed twice with CMF-PBS. The cells were collected into 1.5 mL tubes containing CMF-PBS and then centrifuged at 100× *g* for 5 min at 4 °C. The supernatants were discarded, and 0.5 mL of sonication buffer (20 mM HEPES-NaOH (pH 7.5) and 0.25 M sucrose) was added to the tubes. The cell pellets were homogenized using an ultrasonicator (NR-50M, Microtec, Chiba, Japan). To measure total intracellular tellurium, 0.1 mL of the homogenate was used, and 0.3 mL was further centrifuged at 1000× *g* for 7 min at 4 °C for cell fractionation. The supernatant was collected, and the pellet was defined as the nuclear fraction. The collected supernatant was centrifuged at 2000× *g* for 30 min at 4 °C. The supernatant (1000× *g*) was collected, and the pellet was defined as the mitochondrial fraction. The supernatant (2000× *g*) was further centrifuged at 105,000× *g* for 60 min at 4 °C. The supernatant and pellet were defined as the microsomal and cytosolic fractions, respectively. The pellets obtained in each step were suspended in sonication buffer (0.5 mL), and 0.1 mL of each solution was used for quantification of tellurium. The tellurium content in each fraction was expressed as a percentage of the total fraction.

### 4.5. Ab Initio Quantum Chemical Calculation

First-principles calculations were performed using the General Atomic and Molecular Electronic Structure System (GAMESS version = 1 May 2012 (R1) From Iowa State University) [11]. The exchange-correlation energy was evaluated using the hybrid-GGA B3LYP [24]. The scalar relativistic pseudopotential method was employed with the Huzinaga model core potential (MCP) [25], and the wave functions were expanded by GTO [26] with DZp [27,28,29,30,31]. To take into account the effect of the solvent, the polarizable continuum model (PCM) was used, assuming water with a relative permeability of 78.39.

We describe some details of the calculation on the electron transfer as follows:

(PhM)_2_ + e^−^ → [(PhM)_2_]^•^^−^   (M = S, Se, Te) (5)

The electron enters the anti-bonding state of the M-M bond, and the M-M bond length is elongated by as much as 30%. The electron-transfer process can be described using the M–M distance, *d*, as the reaction coordinate. The total energies of the neutral molecule and the radical anion molecule are denoted as *E*(*d*, (PhM)_2_) and *E*(*d*, [(PhM)_2_]^• −^), respectively, which are calculated when *d* is fixed and other structural parameters are optimized. The energy of the electron is denoted as the chemical potential, *μ*. Starting with the stable structure (*d* = *d*_0_), as d increases gradually, the energy difference between the neutral and radical anion molecules increases and reaches *μ* at *d* = *d*_T_, where the electron can transfer to the molecule while conserving the energy:

(6) $$E\left(d_{T},{\left[(PhM{)}_{2}\right]}^{•-}\right)-E\left(d_{T},(PhM{)}_{2}\right)=\mu$$

The electron transfer needs an activation energy:

(7) $$\Delta E=E\left(d_{T},(PhM{)}_{2}\right)-E\left(d_{0},(PhM{)}_{2}\right)$$

Figure S1 shows the reaction energy diagram for the electron transfer. The activation energy decreased in the order of (a) S, (b) Se, and (c) Te. The energy of the radical anion molecule at *d* = *d*_0_, *E*_0_−, is useful as an index for understanding the order. Table S2 shows that *E*_0_− decreases for a larger *d*_0_. Since the space accepting the extra electron is larger, therefore, *E*_0_− becomes lower. Then, the transition point, *d*_T_, can be closer to *d*_0_, and Δ*E* decreases. We have the rule that a larger M atom needs a lower activation energy for electron transfer to (PhM)_2_ (M = S, Se, Te). From Equations (6) and (7), we obtain Δ*E* as a function of *μ*. Figure S2 shows that this rule can be applied for a wide range of *μ* values.

### 4.6. Statistical Analysis

Data were analyzed for statistical significance by analysis of variance (ANOVA) and Bonferroni’s multiple *t*-test, as appropriate. Statistical significance was set at *p* < 0.05.

## 5. Conclusions

**Figure 5:**
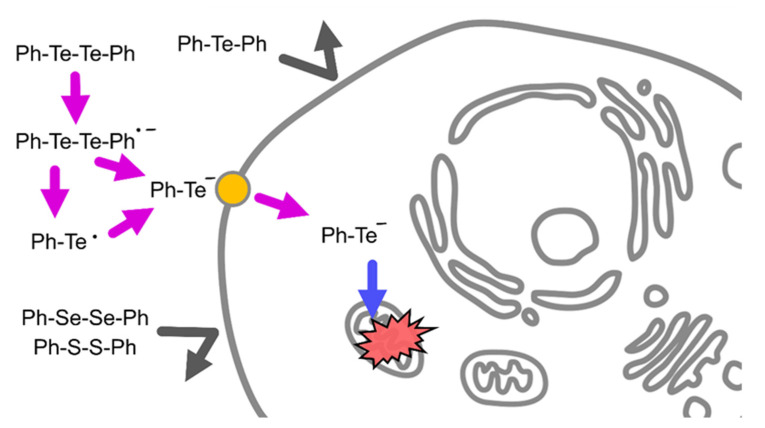
Summary of this study. DPDTe acquires electrons and forms radical anionic molecules. Heterolytic cleavage of the Te–Te bond occurs and produces PhTe^−^. PhTe^−^ is taken up through cell-type dependent mechanisms and translocated to mitochondria. Accumulated PhTe^−^ in the mitochondria induces cell dysfunction and cytotoxicity.

In the present study, it was revealed that DPDTe exhibits cytotoxicity in vascular endothelial cells and fibroblastic IMR-90 cells. The electronic state of tellurium atoms in DPDTe plays an important role in the accumulation and distribution of DPDTe in cultured cells (Figure 5).

## Supplementary Materials

All data are available online at https://www.mdpi.com/article/10.3390/ijms221910520/s1.
