# Supplementary material for: Effects of Substitution on Cytotoxicity of Diphenyl Ditelluride in Cultured Vascular Endothelial Cells
Source: Int J Mol Sci. 2021 Sep 29;22(19):10520. doi: 10.3390/ijms221910520 (PMC8531998; doi:10.3390/ijms221910520)
Supplement: Supplementary file 1 [file ijms-22-10520-s001.zip › supplementary file.pptx]

## Slide 1
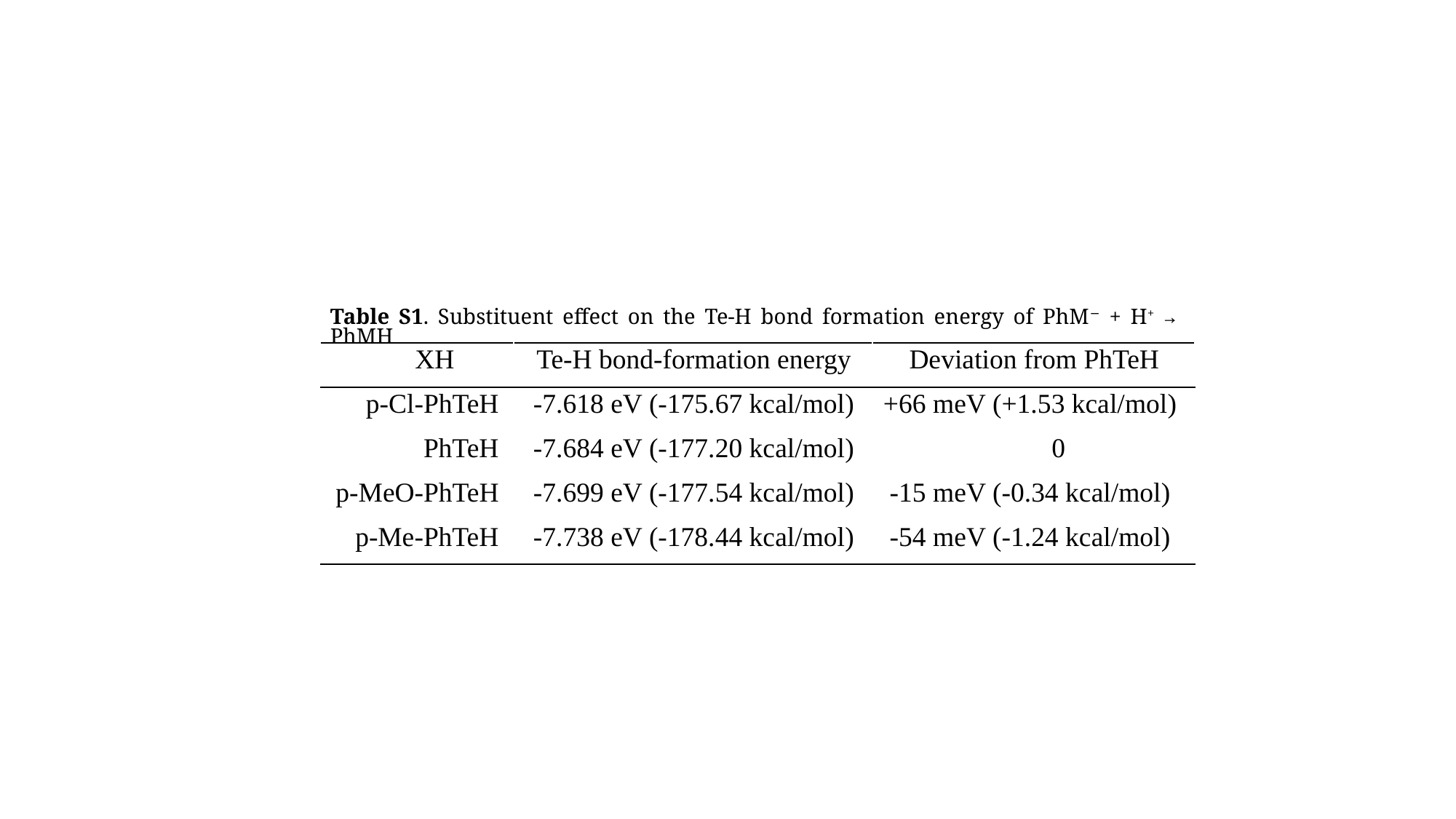

Table S1. Substituent effect on the Te-H bond formation energy of PhM− + H+ → PhMH
| XH | Te-H bond-formation energy | Deviation from PhTeH |
| --- | --- | --- |
| p-Cl-PhTeH | -7.618 eV (-175.67 kcal/mol) | +66 meV (+1.53 kcal/mol) |
| PhTeH | -7.684 eV (-177.20 kcal/mol) | 0 |
| p-MeO-PhTeH | -7.699 eV (-177.54 kcal/mol) | -15 meV (-0.34 kcal/mol) |
| p-Me-PhTeH | -7.738 eV (-178.44 kcal/mol) | -54 meV (-1.24 kcal/mol) |

## Slide 2
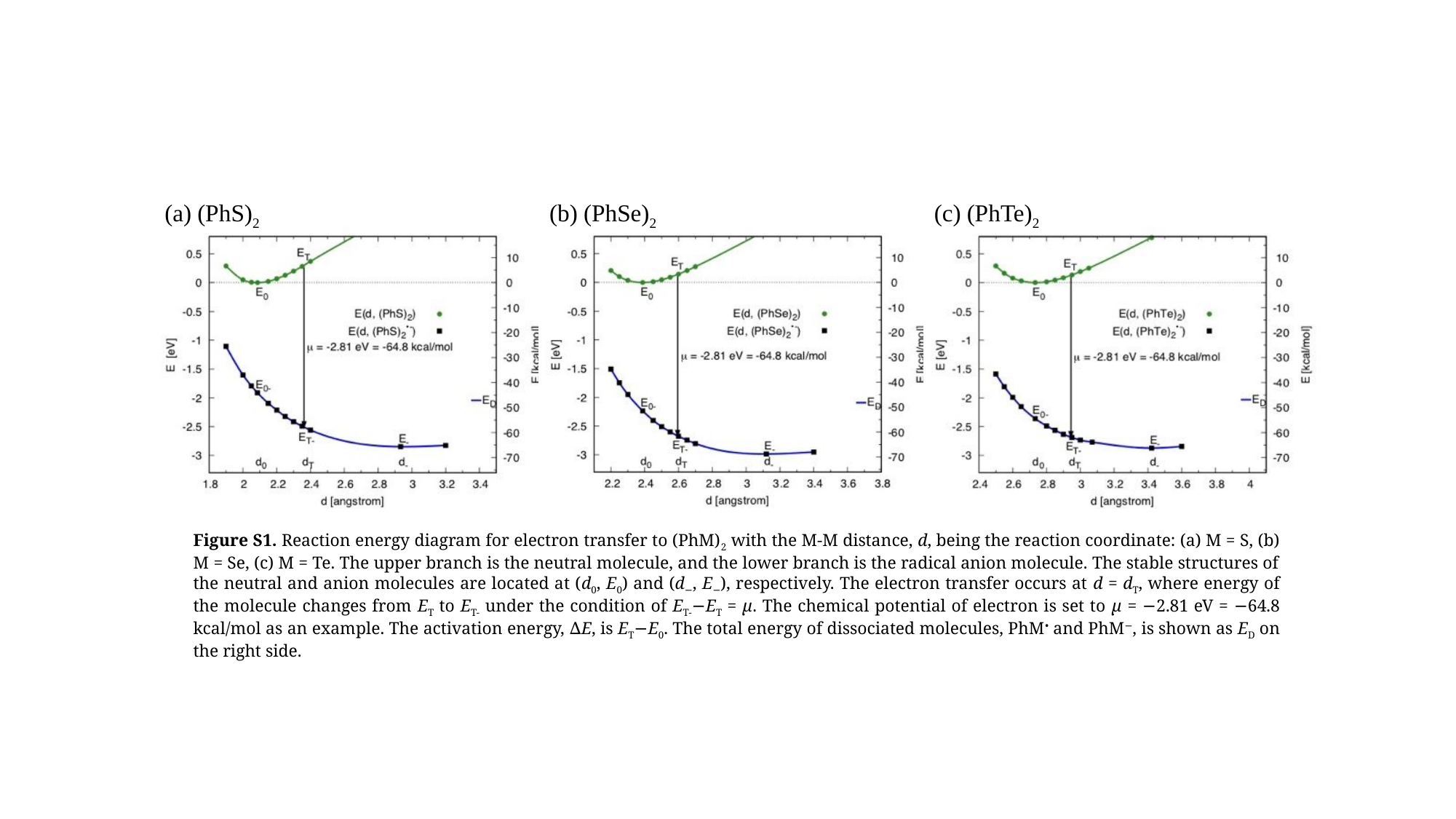

(a) (PhS)2
(b) (PhSe)2
(c) (PhTe)2
Figure S1. Reaction energy diagram for electron transfer to (PhM)2 with the M-M distance, d, being the reaction coordinate: (a) M = S, (b) M = Se, (c) M = Te. The upper branch is the neutral molecule, and the lower branch is the radical anion molecule. The stable structures of the neutral and anion molecules are located at (d0, E0) and (d−, E−), respectively. The electron transfer occurs at d = dT, where energy of the molecule changes from ET to ET- under the condition of ET-−ET = μ. The chemical potential of electron is set to μ = −2.81 eV = −64.8 kcal/mol as an example. The activation energy, ∆E, is ET−E0. The total energy of dissociated molecules, PhM• and PhM−, is shown as ED on the right side.

## Slide 3
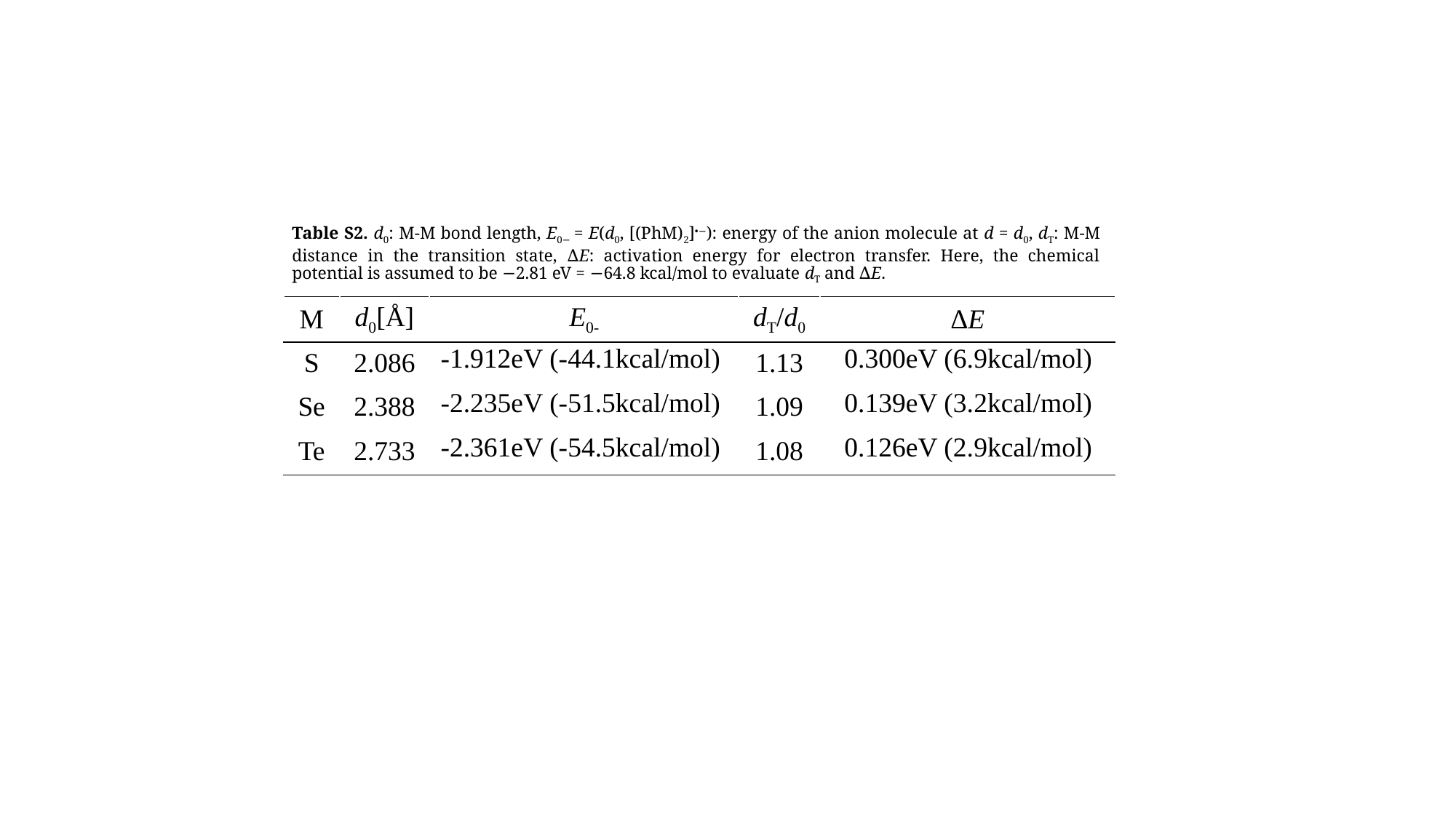

Table S2. d0: M-M bond length, E0− = E(d0, [(PhM)2]•−): energy of the anion molecule at d = d0, dT: M-M distance in the transition state, ∆E: activation energy for electron transfer. Here, the chemical potential is assumed to be −2.81 eV = −64.8 kcal/mol to evaluate dT and ∆E.
| M | d0[Å] | E0- | dT/d0 | ΔE |
| --- | --- | --- | --- | --- |
| S | 2.086 | -1.912eV (-44.1kcal/mol) | 1.13 | 0.300eV (6.9kcal/mol) |
| Se | 2.388 | -2.235eV (-51.5kcal/mol) | 1.09 | 0.139eV (3.2kcal/mol) |
| Te | 2.733 | -2.361eV (-54.5kcal/mol) | 1.08 | 0.126eV (2.9kcal/mol) |

## Slide 4
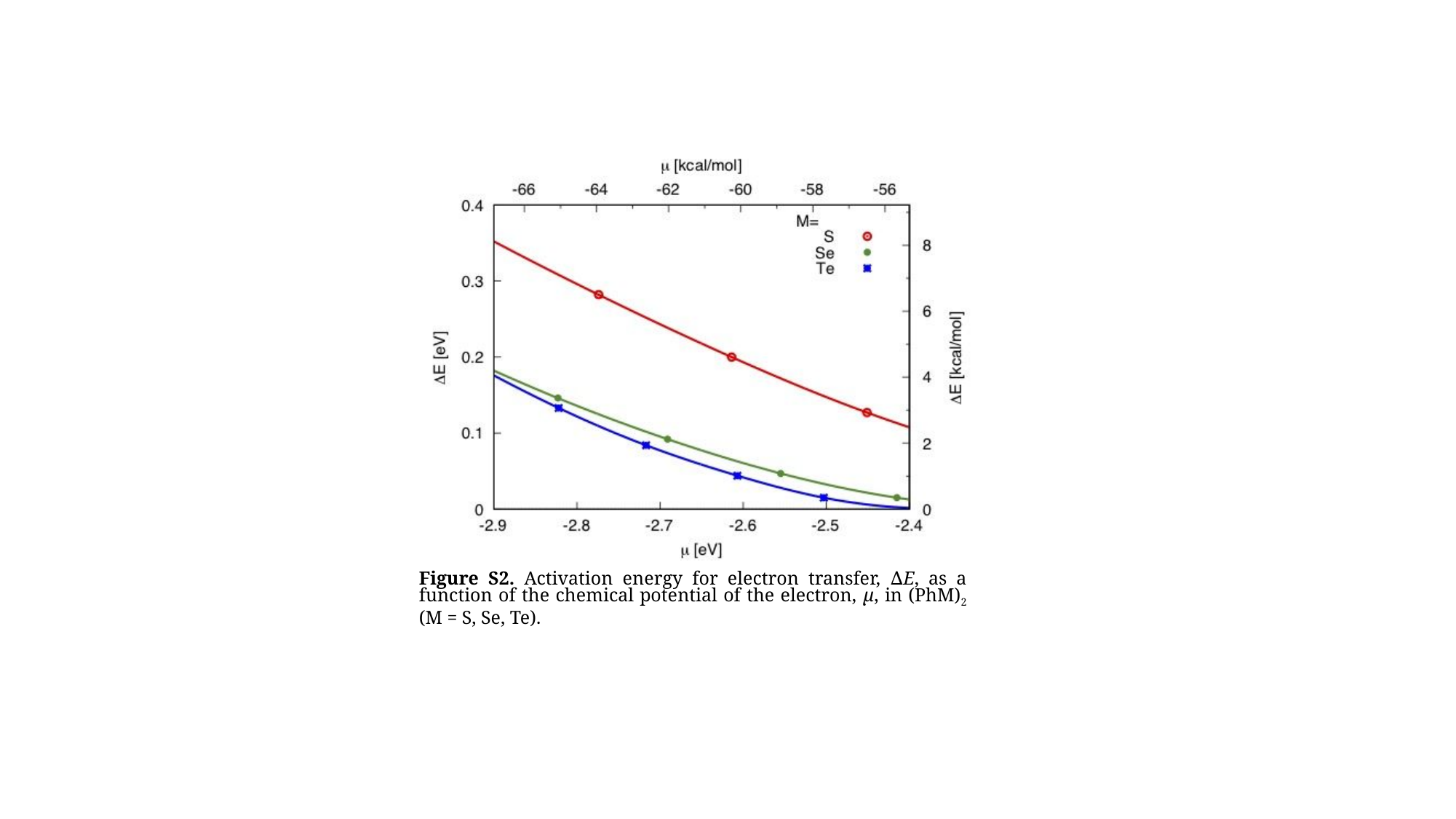

Figure S2. Activation energy for electron transfer, ∆E, as a function of the chemical potential of the electron, μ, in (PhM)2 (M = S, Se, Te).
